# Prospective observational study of the efficacy of oral uracil and tegafur plus leucovorin for stage II colon cancer with risk factors for recurrence using propensity score matching (JFMC46-1201)

**DOI:** 10.1186/s12885-022-09267-z

**Published:** 2022-02-15

**Authors:** Sotaro Sadahiro, Kazuhiro Sakamoto, Takashi Tsuchiya, Takao Takahashi, Hiroki Ohge, Toshihiko Sato, Ken Kondo, Yutaka Ogata, Hideo Baba, Michio Itabashi, Masataka Ikeda, Madoka Hamada, Kiyoshi Maeda, Hiroyuki Masuko, Keiichi Takahashi, Junichi Sakamoto, Mitsuo Kusano, Ichinosuke Hyodo, Masataka Taguri, Satoshi Morita

**Affiliations:** 1 Department of Surgery, Tokai University, 143 Shimokasuya, Isehara, Kanagawa 259-1193 Japan; 2 Department of Coloproctological Surgery, Juntendo University, 2-1-1 Hongo, Bunkyo-ku, Tokyo, 113-8421 Japan; 3 Department of Surgery, Sendai City Medical Center, 5-22-1 Tsurugaya, Miyagino-ku, Sendai, Miyagi, 983-0824 Japan; 4 Department of Digestive Surgery, Gifu University Hospital, 1-1 Yanagido, Gifu, 501-1194 Japan; 5 Department of Infectious Diseases, Hiroshima University Hospital, 1-2-3 Kasumi, Minami-ku, Hiroshima, 734-8551 Japan; 6 Department of Surgery, Yamagata Prefectural Central Hospital, 1800 Aoyagi, Yamagata, 990-2292 Japan; 7 Department of Surgery, Nagoya Medical Center, 4-1-1 Sannomaru, Naka-ku, Nagoya, Aichi 460-0001 Japan; 8 Department of Surgery, Kurume University Hospital Cancer Center, 67 Asahi-machi, Kurume, Fukuoka, 830-0011 Japan; 9 Department of Gastroenterological Surgery, Kumamoto University, 1-1-1 Honjo, Chuo-ku, Kumamoto, 860-8556 Japan; 10 Department of Surgery, Division of Inflammatory Bowel Disease Surgery, Tokyo Women’s Medical University, 8-1 Kawada-cho, Shinjuku-ku, Tokyo, 162-8666 Japan; 11 Department of Gastroenterological Surgery, Hyogo College of Medicine, 1-1 Mukogawa-cho, Nishinomiya, Hyogo 663-8501 Japan; 12 Division of Gastrointestinal Surgery, Kansai Medical University Hospital, 2-3-1 Shinmachi Hirakata, Osaka, 573-1191 Japan; 13 Department of Gastroenterological Surgery, Osaka City General Hospital, 2-13-22 Miyakojimahondori, Miyakojima-ku, Osaka, 534-0021 Japan; 14 Department of Surgery, Nikko Memorial Hospital, 1-5-13 Shintomi-cho, Muroran, Hokkaido, 051-8501 Japan; 15 Tokyo Metropolitan Health and Hospitals Corporation Ohkubo Hospital, 2-44-1 Kabuki-cho, Shinjuku-ku, Tokyo, 160-8488 Japan; 16 Tokai Central Hospital, 4-6-2 Sohara Higashijima-cho, Kakamigahara, Gifu, 504-8601 Japan; 17 Department of Physical Medicine, Yoichi Hospital, 19-1-1 Kurokawa-cho Yoichi, Hokkaido, 046-0003 Japan; 18 Department of Gastrointestinal Medical Oncology, National Hospital Organization Shikoku Cancer Center, 160 Kou, Minamiumemoto, Matsuyama, Ehime 791-0280 Japan; 19 Department of Data Science, Yokohama City University, 22-2 Seto, Kanazawa-ku, Yokohama, Kanagawa 236-0027 Japan; 20 Department of Biomedical Statistics and Bioinformatics, Kyoto University, 54 Kawahara-cho, Shogoin, Sakyo-ku, Kyoto, 606-8507 Japan

**Keywords:** Adjuvant chemotherapy, Colon cancer, High-risk, Stage II, Propensity score, Risk factor, Tegafur, Uracil, Leucovorin, Inverse probability of treatment weighting

## Abstract

**Background:**

The efficacy of adjuvant chemotherapy for high-risk stage II colon cancer (CC) has not been well established. We compared the effects of surgery with and without oral uracil and tegafur plus leucovorin (UFT/LV) in patients with high-risk stage II CC, adjusting for potential risk factors.

**Methods:**

We enrolled patients with histologically confirmed stage II colon adenocarcinoma with at least one of the following conditions: T4 disease, perforation/penetration, poorly differentiated adenocarcinoma/mucinous carcinoma, or < 12 dissected lymph nodes. Patients chose to be non-randomized or randomized to undergo surgery alone (NR-Group S or R-Group S) or surgery followed by 6 months of UFT/LV (NR-Group U or R-Group U). The primary endpoint was disease-free survival (DFS) after adjusting for previously reported risk factors using propensity score matching (1:2) and inverse probability of treatment weighting (IPTW) in the non-randomized arm.

**Results:**

Overall, 1,902 (98%) and 36 (2%) patients were enrolled in the non-randomized and randomized arms, respectively. There were too few patients in the randomized arm and these were therefore excluded from the analysis. Of the 1,902 patients, 402 in NR-Group S and 804 in NR-Group U were propensity score-matched. The 3-year DFS rate (95% confidence interval) was significantly higher in NR-Group U (80.9% [77.9%–83.4%]) than in NR-Group S (74.0% [69.3%–78.0%]) (hazard ratio, 0.64 [0.50–0.83]; *P* = 0.0006). The 3-year overall survival rate was not significantly different between NR-Group S and NR-Group U. Significantly higher 3-year DFS (*P* = 0.0013) and overall survival (*P* = 0.0315) rates were observed in NR-Group U compared with NR-Group S using IPTW.

**Conclusions:**

Adjuvant chemotherapy with UFT/LV showed a significant survival benefit over surgery alone in patients with high-risk stage II CC characterized by at least one of the following conditions: T4 disease, perforation/penetration, poorly differentiated adenocarcinoma/mucinous carcinoma, or < 12 dissected lymph nodes.

**Trial registration:**

Japan Registry of Clinical Trials: jRCTs031180155 (date of registration: 25/02/2019) (UMIN Clinical Trials Registry: UMIN000007783, date of registration: 18/04/2012).

**Supplementary Information:**

The online version contains supplementary material available at 10.1186/s12885-022-09267-z.

## Background

Colorectal cancer (CRC) is the most frequently diagnosed cancer and the second leading cause of cancer-related death, accounting for > 50,000 cases in Japan in 2018 [1]. Clinical guidelines from the Japanese Society for Cancer of the Colon and Rectum advise against routine administration of adjuvant chemotherapy to all patients with stage II CRC,^1^ but the American Society of Clinical Oncology, the National Comprehensive Cancer Network, and the European Society for Medical Oncology suggest that adjuvant chemotherapy is beneficial in patients with stage II colon cancer (CC) with high-risk factors for recurrence [2–5]. However, unlike the proven benefits of adjuvant chemotherapy for patients with stage III CC, the benefits for patients with stage II CC remain unclear because of an absence of conclusive data from randomized controlled clinical trials [6, 7]. In addition, risk factors for stage II CC have not been fully established [2, 5, 8–12].

Large-scale randomized comparative studies are challenging, especially when they include a surgery-alone arm, because risk factors for stage II CC have not been established and effective evidence-based adjuvant chemotherapies are lacking. To overcome these issues, we conducted a multi-center prospective two-arm trial, including a non-randomized arm, in which patients chose whether to undergo surgery alone or surgery followed by oral uracil and tegafur plus leucovorin (UFT/LV) with propensity score (PS) matching [13, 14] to minimize confounding biases in between-group comparisons, and a randomized arm, in which patients chose to be assigned randomly to undergo either surgery alone or surgery followed by UFT/LV. Oncologic outcomes and safety were evaluated after adjusting for previously reported clinicopathological risk factors using PS matching and inverse probability of treatment weighting (IPTW) in the non-randomized arm.

## Methods

Details of the study protocol can be found in our previous report [15].

### Patients

Eligible patients were aged 20 to 80 years with histologically confirmed stage II colon adenocarcinoma with at least one of the following conditions: T4 disease, perforation/penetration, poorly differentiated adenocarcinoma/mucinous carcinoma, or < 12 dissected lymph nodes. Eligible patients had undergone R0 resection, had an Eastern Cooperative Oncology Group performance status of 0/1, could take the study drugs orally, and could begin the study treatment within 8 weeks after surgery.

### Allocation to treatment

This prospective observational study was based on patients’ self-selected treatments: the non-randomized arm comprised patients who chose to undergo surgery alone (NR-Group S) or surgery followed by UFT/LV (NR-Group U), while the randomized arm comprised patients who were assigned to undergo surgery alone (R-Group S) or surgery followed by UFT/LV (R-Group U). Patients in the randomized arm were allocated (1:1) using the web-based minimization method and stratified by depth of tumor invasion, histology, number of lymph nodes examined, age, sex, and institution.

### Protocol treatment

Patients in NR-Group S and R-Group S underwent a blood test every 3 months and chest and abdominal computed tomography examinations every 6 months for up to 5 years or until confirmation of recurrence, occurrence of other malignancies, or death. Colonoscopy was also performed 1 and 3 years after surgery. Adverse events (AEs) were monitored for 6 months after registration.

Patients in NR-Group U and R-Group U started treatment with oral UFT (300 mg/m^2^/day) and LV (75 mg/day), three doses per day (every 8 h) within 8 weeks after surgery under one of the following two regimens: daily for 28 days followed by a 7-day rest or daily for 5 days followed by a 2-day rest. One course lasted for 5 weeks, and a total of five courses were given. If the criteria for starting/continuing treatment were not fulfilled, treatment was postponed or suspended until AEs improved sufficiently to meet the criteria. Depending on AE severity, the UFT dose was reduced according to the protocol. Physical examinations and blood tests were performed during the first week of each course, and medication compliance was monitored. Concomitant use of anti-tumor drugs other than UFT/LV, radiation therapy, and immunotherapy for the target disease were prohibited until confirmation of recurrence, double cancer, or multiple primary CRC. After completion of UFT/LV therapy, patients were followed up according to the same schedule used in NR-Group S and R-Group S, except that AEs were monitored from the start of UFT/LV therapy until 28 days after the final dose.

### Endpoints

The primary endpoint of this study was disease-free survival (DFS), which was defined as the time from the date of registration to the date of occurrence of secondary cancer, recurrence, or death of any cause, whichever occurred first. The secondary endpoints were overall survival (OS), which was defined as the time from the date of registration to the date of death of any cause, and the incidences of AEs by severity. Expression of carcinoembryonic antigen mRNA 24 h after surgery was also measured as a secondary endpoint, and the results will be presented in a separate report.

### Statistical analysis

The target sample size in the non-randomized arm with a sample size ratio of 1:2 between NR-Group S and NR-Group U was estimated at 1,715, and the target sample size in the randomized arm was 1,100 (550 patients each in R-Group S and R-Group U) [15].

The efficacy endpoints were evaluated in the full-analysis set, which included all enrolled patients, except those with major protocol violations in the non-randomized and randomized arms. The efficacy endpoints were also evaluated in the PS-matched population, which excluded patients who did not match, in the non-randomized arm. Safety was analyzed in the safety-analysis set, which included all non-randomized or randomized patients, except those who did not receive UFT/LV.

For the analysis of the non-randomized population, 1:2 PS matching was used to minimize confounding biases in comparisons between NR-Group S and NR-Group U [13, 14]. Potential confounding factors for estimating the PS were prespecified as follows: age (≥ 70 or < 70 years), sex, number of dissected lymph nodes (≥ 12 or < 12), T4 disease, bowel perforation, poorly differentiated adenocarcinoma, mucinous carcinoma, and number of participating patients at each institution (≥ 5 or < 5). The caliper for PS matching was determined before the analysis so that the standardized difference of all confounding factors was < 0.1. Differences before and after matching were assessed using the χ^2^ test. A stratified log-rank test accounting for matched pairs was used to compare groups using a two-sided significance level of 5%. Hazard ratios (HRs) and 95% confidence intervals (CIs) were calculated using a stratified Cox proportional hazards model. The Kaplan–Meier method was used to estimate the 3-year DFS and OS rates in each group, and Greenwood’s formula was used to calculate the 95% CIs.

Analyses were also conducted with IPTW using estimated PSs [16]. For the IPTW analysis, standard errors were estimated using the robust sandwich variance estimator [17].

For the analysis of efficacy in the randomized arm, the same analyses were performed with the same stratification factors, except for institution. The incidence of each AE was calculated and the severity was evaluated using the Japanese version of the Common Terminology Criteria for Adverse Events v4.0. All statistical analyses were performed using SAS version 9.4 (SAS Institute, Cary, NC, USA).

## Results

### Patient dispositions and characteristics before and after PS matching

**Fig. 1:**
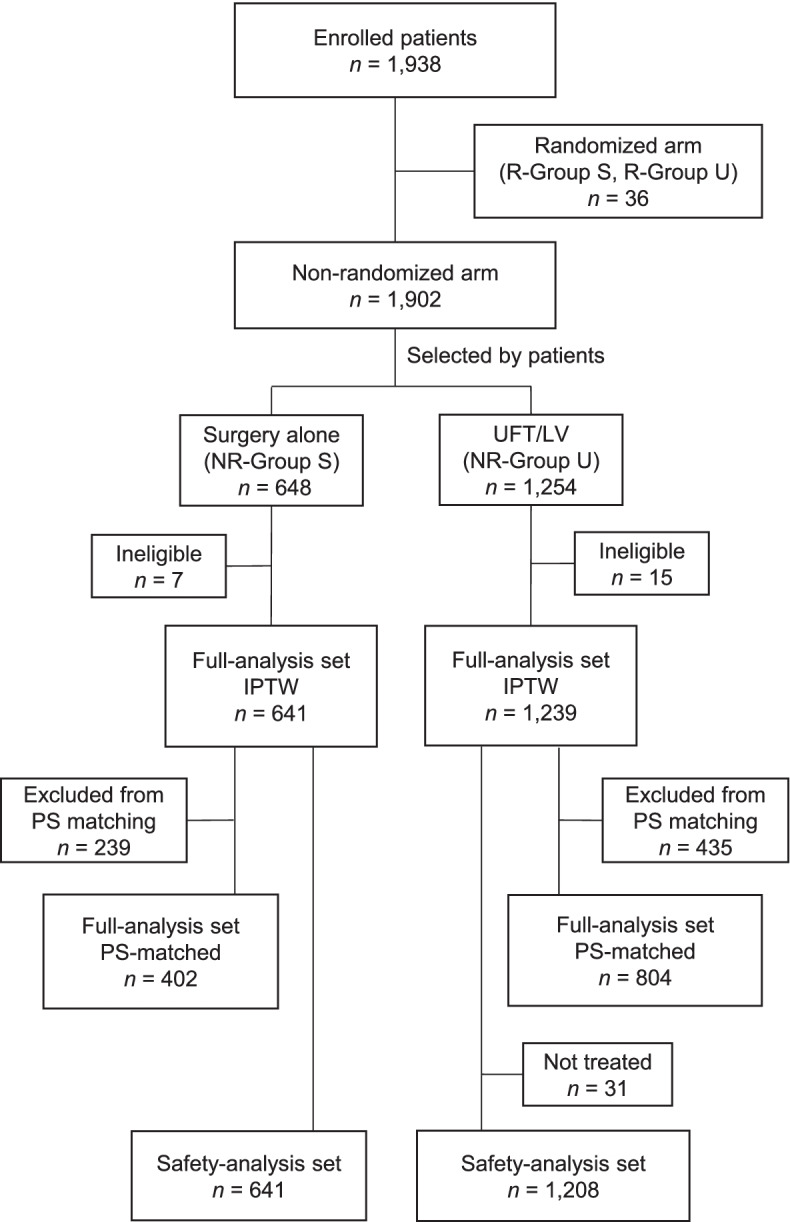
Patient flow diagram. Patients in NR-Group S and R-Group S underwent surgery alone; patients in NR-Group U and R-Group U underwent surgery followed by UFT/LV. Abbreviations: IPTW, inverse probability of treatment weighting; NR, non-randomized; PS, propensity score; R, randomized; UFT/LV, uracil and tegafur plus leucovorin

A total of 1,938 patients were enrolled from 321 institutions in Japan from May 2012 to April 2016, including 1,902 patients (98%) in the non-randomized arm and 36 (2%) in the randomized arm (Fig. 1). The number of patients in the randomized arm was too small and, they were therefore excluded from the following analysis.

The baseline characteristics of the 1,880 eligible patients in the non-randomized arm (641 patients in NR-Group S and 1,239 patients in NR-Group U) are summarized in Table 1.

**Table 1:** Baseline characteristics of patients before and after propensity score matching in the non-randomized arm

	Non-randomized arm						
	Pre-PS matching			Post-PS matching		Standardized difference	
	Surgery only (NR-Group S) *n* = 641	Surgery plus UFT/LV (NR-Group U) *n* = 1,239	Total (NR-Group) *N* = 1,880	Surgery only (NR-Group S) *n* = 402	Surgery plus UFT/LV (NR-Group U) *n* = 804	Pre-PS matching	Post-PS matching
Age^a^, years							
Mean ± SD	69.9 ± 8.1	65.1 ± 9.9	66.7 ± 9.6	66.7 ± 8.5	64.4 ± 9.4	—	—
Median [range]	71 [34–80]	66 [29–80]	68 [29–80]	67 [34–80]	65 [29–80]	—	—
< 70	264 (41.2)	771 (62.2)	1,035 (55.1)	264 (65.7)	555 (69.0)	0.431	0.072
≥ 70	377 (58.8)	468 (37.8)	845 (44.9)	138 (34.3)	249 (31.0)	—	—
Sex^a^, male	369 (57.6)	654 (52.8)	1,023 (54.4)	216 (53.7)	454 (56.5)	0.096	0.055
Risk factors for recurrence^a^							
T4 disease	302 (47.1)	720 (58.1)	1,022 (54.4)	217 (54.0)	460 (57.2)	0.222	0.065
Perforation/penetration	52 (8.1)	148 (11.9)	200 (10.6)	49 (12.2)	116 (14.4)	0.128	0.066
Poorly differentiated adenocarcinoma	46 (7.2)	127 (10.3)	173 (9.2)	39 (9.7)	87 (10.8)	0.109	0.037
Mucinous carcinoma	72 (11.2)	190 (15.3)	262 (13.9)	65 (16.2)	120 (14.9)	0.121	0.034
No. of lymph nodes dissected < 12	298 (46.5)	403 (32.5)	701 (37.3)	149 (37.1)	269 (33.5)	0.289	0.076
No. of patients at institution ≥ 5	549 (85.6)	969 (78.2)	1,518 (80.7)	331 (82.3)	653 (81.2)	0.194	0.029
Performance status							
0	589 (91.9)	1,175 (94.8)	1,764 (93.8)	367 (91.3)	756 (94.0)	0.119	0.105
1	52 (8.1)	64 (5.2)	116 (6.2)	35 (8.7)	48 (6.0)	—	—
Surgical approach							
Laparoscopy	404 (63.0)	675 (54.5)	1,079 (57.4)	237 (59.0)	430 (53.5)	0.174	0.110
Laparotomy	237 (37.0)	564 (45.5)	801 (42.6)	165 (41.0)	374 (46.5)	—	—
Histology							
Papillary and tubular	525 (81.9)	928 (74.9)	1,453 (77.3)	300 (74.6)	602 (74.9)	0.171	0.006
Poorly differentiated and other	116 (18.1)	311 (25.1)	427 (22.7)	102 (25.4)	202 (25.1)	—	—
Depth of tumor invasion (TNM classification)							
T3	339 (52.9)	519 (41.9)	858 (45.6)	185 (46.0)	344 (42.8)	0.222	0.065
T4	302 (47.1)	720 (58.1)	1,022 (54.4)	217 (54.0)	460 (57.2)	—	—
Lymph node dissection							
Mean ± SD	17.9 ± 13.3	21.0 ± 14.3	20.0 ± 14.0	19.9 ± 14.0	20.7 ± 13.7	0.228	0.055
Median [range]	13 [1–107]	18 [1–129]	17 [1–129]	16 [1–107]	18 [1–89]	—	—
Lymphatic invasion							
Ly0	285 (44.5)	501 (40.4)	786 (41.8)	170 (42.3)	321 (39.9)	0.081^b^	0.048
≥ Ly1	356 (55.5)	737 (59.5)	1,093 (58.1)	232 (57.7)	483 (60.1)	—	—
Missing	0 (0.0)	1 (0.1)	1 (0.1)	0 (0.0)	0 (0.0)	—	—
Venous invasion							
V0	235 (36.7)	400 (32.3)	635 (33.8)	149 (37.1)	256 (31.8)	0.092^b^	0.110
≥ V1	406 (63.3)	838 (67.6)	1,244 (66.2)	253 (62.9)	548 (68.2)	—	—
Missing	0 (0.0)	1 (0.1)	1 (0.1)	0 (0.0)	0 (0.0)	—	—
Tumor location							
Right colon (C, A, T)	253 (39.5)	550 (44.4)	803 (42.7)	167 (41.5)	355 (44.2)	0.100	0.053
Left colon (D, S, RS)	388 (60.5)	689 (55.6)	1,077 (57.3)	235 (58.5)	449 (55.8)	—	—

Values presented as *n* (%) unless otherwise specified. NR-Group S: patients underwent surgery alone; NR-Group U: patients underwent surgery followed by UFT/LV

^a^Used for PS matching

^b^Missing data excluded from calculation

—: Not applicable

*Abbreviations*: *A* Ascending colon, *C* Cecum, *D* Descending colon, *NR* Non-randomized, *PS* Propensity score, *RS* Rectosigmoid colon, *S* Sigmoid colon, *SD* Standard deviation, *T* Transverse colon, *TNM* Tumor, node, metastasis, *UFT/LV* Uracil sand tegafur plus leucovorin

After 1:2 PS matching, 402 patients in NR-Group S and 804 patients in NR-Group U were matched, with no significant differences in the eight confounding factors between the groups (Table 1). The standardized difference for all confounding factors was < 0.08.

### Survival outcomes in the PS-matched population

**Fig. 2:**
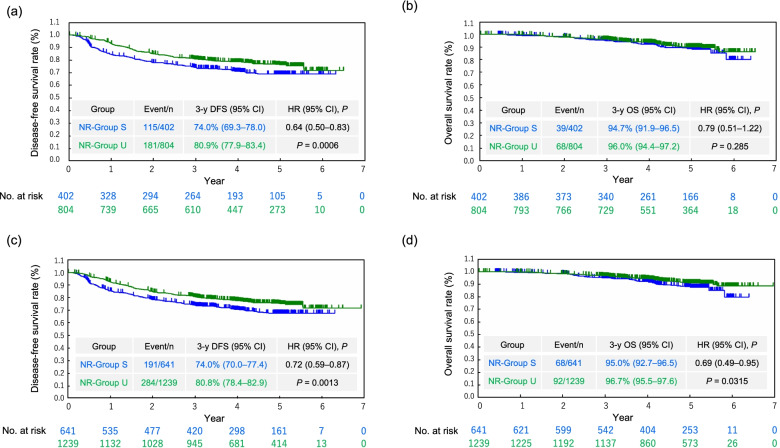
Disease-free and overall survival in non-randomized patients with resected high-risk stage II colon cancer. **a** Disease-free and **b** overall survival in the propensity score-matched groups. **c** Disease-free and **d** overall survival in the inverse probability of treatment weighting groups. NR-Group S (blue): surgery alone; NR-Group U (green): surgery followed by UFT/LV treatment. Abbreviations: CI, confidence interval; DFS, disease-free survival; HR, hazard ratio; NR, non-randomized; OS, overall survival; UFT/LV, uracil and tegafur plus leucovorin. *P* values obtained by log-rank test

As of the data cut-off date on December 11, 2019, the median follow-up time for DFS in NR-Groups S and U was 59.0 months (interquartile range [IQR], 47.2–60.6 months) in the PS-matched population. The 3-year DFS rate was significantly higher in NR-Group U (80.9% [77.9%–83.4%]) than in NR-Group S (74.0% [69.3%–78.0%]) (HR, 0.64 [0.50–0.83]; *P* = 0.0006) (Fig. 2a and Supplementary Table S1). The median follow-up time for OS in NR-Group S and U was 59.8 months (IQR, 47.5–61.2 months) in the PS-matched population. No significant difference was observed in the 3-year OS rate between NR-Group S and NR-Group U (*P* > 0.2) (Fig. 2b and Supplementary Table S1).

### Events in the PS-matched population

The median time to recurrence was significantly longer in NR-Group U (17.2 months; range, 1.8–59.8 months) than in NR-Group S (8.7 months; range, 2.0–47.1 months) (*P* < 0.0001). There were 115 (28.6%) and 181 (22.5%) DFS events in NR-Group S and NR-Group U, respectively. Recurrence of primary CC occurred in 73 patients (18.2%) in NR-Group S (lung and liver were the most common recurrence sites) and in 123 patients (15.3%) in NR-Group U (peritoneum and liver were the most common recurrence sites). There were 39 (9.7%) and 68 (8.5%) deaths in NR-Group S and NR-Group U, respectively (Table 2).

**Table 2:** Summary of disease-free survival and overall survival events

	Non-randomized arm	
	Surgery only (NR-Group S) *n* = 402	Surgery plus UFT/LV (NR-Group U) *n* = 804
**Disease-free survival events**^**a**^	115 (28.6)	181 (22.5)
Recurrence	73 (18.2)	123 (15.3)
Sites (multiple selections made)		
Liver	24 (6.0)	37 (4.6)
Lung	25 (6.2)	29 (3.6)
Local	12 (3.0)	25 (3.1)
Anastomosis	5 (1.2)	13 (1.6)
Regional lymph node	0 (0.0)	3 (0.4)
Other	7 (1.7)	12 (1.5)
Lymph nodes other than regional lymph nodes	5 (1.2)	8 (1.0)
Peritoneum	17 (4.2)	40 (5.0)
Other	4 (1.0)	11 (1.4)
Uterus	0 (0.0)	1 (0.1)
Ovary	2 (0.5)	3 (0.4)
Other	2 (0.5)	7 (0.9)
Secondary cancer	39 (9.7)	50 (6.2)
Death	3 (0.7)	8 (1.0)
** Overall survival events**	39 (9.7)	68 (8.5)

Values presented as *n* (%)

^a^The first disease-free survival event experienced by each patient was exhibited. NR-Group S: patients underwent surgery alone; NR-Group U: patients underwent surgery followed by UFT/LV

Abbreviations: *NR* Non-randomized, *UFT/LV* Uracil and tegafur plus leucovorin

### Survival outcomes in the IPTW population

The patients’ background characteristics were well balanced between NR-Group S (*n* = 641) and NR-Group U (*n* = 1,239) (Table 1). The median follow-up time for DFS was 58.9 months (IQR, 47.2–60.6 months) and the 3-year DFS rate (95% CI) was significantly higher in NR-Group U than in NR-Group S (*P* = 0.0013) (Fig. 2c and Supplementary Table S1). The median follow-up time for OS was 59.8 months (IQR, 47.5–61.2 months) and the 3-year OS rate was significantly higher in NR-Group U than in NR-Group S (*P* = 0.0315) (Fig. 2d and Supplementary Table S1).

## Treatment with UFT/LV and safety

Of 1,239 patients in NR-Group U in the IPTW population, 890 (71.8%) completed UFT/LV treatment, 318 (25.7%) discontinued treatment, and 31 (2.5%) did not receive treatment. Treatment discontinuation occurred most frequently during the first course (140/1,208, 11.6%), and the most common reason for discontinuation was AEs (209/318, 65.7%). Of 1,208 patients in NR-Group U, 1,040 (86.1%) were treated without decreasing the doses of the study drugs.

Grade ≥ 3 AEs that occurred with a frequency of ≥ 2% in NR-Group U were diarrhea (3.9%), elevated alanine aminotransferase (3.1%), and elevated aspartate aminotransferase (2.2%). No other notable AEs with respect to incidence or severity occurred in any group (Supplementary Table S2).

## Discussion

Studies involving patients with stage II/III or III CC have shown that 5-fluorouracil with or without oxaliplatin or LV, UFT/LV, and capecitabine are effective adjuvant chemotherapies. However, the efficacies of these therapies in patients with stage II CC are inconsistent [4, 18–22]. UFT/LV is widely used in Japan because it can be administered orally and has shown similar efficacy and safety to intravenous 5-fluorouracil/LV in patients with stage III disease [18, 23].

Consistent with our previous study demonstrating that 6 months of oral UFT/LV was sufficient in patients with stage IIB/III CC [24], the current prospective observational study demonstrated a significant 3-year DFS benefit following 6 months of oral UFT/LV compared with surgery alone in patients with resected high-risk stage II CC, adjusted using PS matching.

A previous study showed that the time to recurrence after curative resection of CC was significantly longer in patients treated with adjuvant chemotherapy than in patients treated with surgery alone [25]. When the incidence rate of DFS events was presented in 3-month intervals, the incidence of DFS events was highest at 6 to 9 months after registration in patients who underwent surgery alone; however, this peak level was lower in patients treated with UFT/LV, indicating that 6 months of UFT/LV effectively suppressed the peak of DFS events (Supplementary Fig. S1).

We found no significant difference in OS between NR-Group S and NR-Group U. However, using IPTW, UFT/LV therapy significantly improved OS. This improvement may have been detected because of the higher statistical power provided by the inclusion of all patients (*n* = 1,880). However, patients in the PS-matched population require long-term observation (5–10 years) before conclusions can be drawn regarding the survival benefits of UFT/LV.

This study included non-randomized and randomized arms because of potential difficulties in obtaining sufficient numbers of patients willing to participate in a randomized study without reliable measures to estimate the risk of recurrence, and because of the use of a therapy with no evidence-based effectiveness or safety for stage II disease. As predicted during preparation of the study protocol, 98% of the 1,938 registered patients self-selected their treatment. Among the patients who chose their treatment, 34% selected surgery alone and 66% chose surgery followed by adjuvant chemotherapy. Our results are consistent with a previous study in which > 60% of patients with CC answered a questionnaire saying that they would undergo adjuvant chemotherapy if the treatment reduced the 5-year recurrence rate by 1% to 2% [26].

The incidence rates of AEs in the present study were lower than those previously reported, and the completion rate of UFT/LV treatment was similar to equivalent and similar regimens available for patients with the same stage of and/or more-advanced CC [25, 27]. Six months of oral UFT/LV therapy thus appeared to be safe and well-tolerated as a postoperative adjuvant chemotherapy.

In this study, we applied PS matching to the non-randomized arm to adjust for confounding variables to assess the effects of UFT/LV. Increasing numbers of studies have used this analysis, and we showed that it was indeed a useful statistical method for achieving similar distributions of observed baseline covariates. We therefore expect that the data obtained from meta-analyses and non-randomized controlled studies can be analyzed using PS matching to provide statistically and clinically meaningful information.

This study had some limitations. The main results were obtained from a non-randomized population. Although PS matching and IPTW were used to adjust for risk factors, we cannot exclude other unknown confounding factors that might have caused a certain degree of bias. In addition, some risk factors, such as microsatellite instability [28, 29], were not included. We also excluded lymphatic, vascular, or perineural invasion as a risk factor because its evaluation was likely to be inconsistent among the participating institutions. Further studies are therefore required to validate the present results with respect to the risk factors used for adjustment.

## Conclusions

The present results suggest that 6 months of adjuvant chemotherapy with oral UFT/LV has significant survival benefits in patients with stage II CC characterized by at least one of the following conditions: T4 disease, perforation/penetration, poorly differentiated adenocarcinoma/mucinous carcinoma, or < 12 dissected lymph nodes.

## Supplementary Information

**Additional file 1: Supplementary Table S1.** Summary of survival endpoints in non-randomized arm. **Supplementary Table S2.** Incidences of grade ≥3 adverse events. **Supplementary Figure S1.** Incidence rate of disease-free survival events at 3-month intervals in propensity score-matched non-randomized patients with resected high-risk stage II colon cancer.

## Data Availability

The datasets used and/or analyzed during the current study are available from the corresponding author on reasonable request.

## Abbreviations

CC: Colon cancer
UFT: Uracil and tegafur
LV: Leucovorin
NR: Non-randomized
R: Randomized
DFS: Disease-free survival
IPTW: Inverse probability of treatment weighting
CRC: Colorectal cancer
PS: Propensity score
AE: Adverse events
OS: Overall survival
HR: Hazard ratio
CI: Confidence interval
IQR: Interquartile range
