# Supplementary material for: Prospective observational study of the efficacy of oral uracil and tegafur plus leucovorin for stage II colon cancer with risk factors for recurrence using propensity score matching (JFMC46-1201)
Source: BMC Cancer. 2022 Feb 15;22:170. doi: 10.1186/s12885-022-09267-z (PMC8845390; doi:10.1186/s12885-022-09267-z)
Supplement: Supplementary file 1 — Additional file 1: Supplementary Table S1. Summary of survival endpoints in non-randomized arm. Supplementary Table S2. Incidences of grade ≥3 adverse events. Supplementary Figure S1. Incidence rate of disease-free survival events at 3-month intervals in propensity score-matched non-randomized patients with resected high-risk stage II colon cancer. [file 12885_2022_9267_MOESM1_ESM.pdf]

Supplementary information

Supplementary Table S1. Summary of survival endpoints in non-randomized arm.

Survival	Analysis population	Groups compared	Stratification factor	Weighting	X^2 value	*P*-value
DFS	FAS<br>PS matching	NR-Group<br>S vs. U	Matched pair		11.84	0.0006
DFS	FAS	NR-Group<br>S vs. U		IPTW	11.41	0.0013
OS	FAS<br>PS matching	NR-Group<br>S vs. U	Matched pair		1.14	0.285
OS	FAS	NR-Group<br>S vs. U		IPTW	4.63	0.0315

DFS and OS calculated from date of registration. NR-Group S: patients underwent surgery alone; NR-Group U: patients underwent surgery followed by UFT/LV.

Abbreviations: DFS, disease-free survival; FAS, full-analysis set; IPTW, inverse probability of treatment weighting; NR, non- randomized; OS, overall survival; PS, propensity score; UFT/LV, uracil and tegafur plus leucovorin.

Supplementary Table S2. Incidences of grade ≥3 adverse events.

	All AEs		Grade ≥3 AEs	
	Surgery only (NR-Group S) *n* = 641	UFT/LV (NR-Group U) *n* = 1,208	Surgery only (NR-Group S) *n* = 641	UFT/LV (NR-Group U) *n* = 1,208
Leukopenia	16 (2.5)	145 (12.0)	0 (0.0)	3 (0.2)
Neutropenia	10 (1.6)	130 (10.8)	0 (0.0)	8 (0.7)
Thrombocytopenia	17 (2.7)	83 (6.9)	1 (0.2)	1 (0.1)
Hemoglobin decreased	74 (11.5)	268 (22.2)	2 (0.3)	7 (0.6)
Aspartate aminotransferase increased	27 (4.2)	200 (16.6)	1 (0.2)	26 (2.2)
Alanine aminotransferase increased	24 (3.7)	204 (16.9)	0 (0.0)	38 (3.1)
Blood bilirubin increased	20 (3.1)	201 (16.6)	1 (0.2)	6 (0.5)
Creatinine increased	28 (4.4)	40 (3.3)	0 (0.0)	2 (0.2)
Nausea	3 (0.5)	157 (13.0)	0 (0.0)	12 (1.0)
Vomiting	0 (0.0)	49 (4.1)	0 (0.0)	3 (0.2)
Diarrhea	4 (0.6)	261 (21.6)	0 (0.0)	47 (3.9)
Decreased appetite	2 (0.3)	191 (15.8)	0 (0.0)	21 (1.7)
Fatigue	1 (0.2)	140 (11.6)	0 (0.0)	6 (0.5)
Skin hyperpigmentation	0 (0.0)	59 (4.9)	0 (0.0)	0 (0.0)
Mucositis oral	0 (0.0)	102 (8.4)	0 (0.0)	5 (0.4)

Data presented as n (%). NR-Group S: patients underwent surgery alone; NR-Group U: patients underwent surgery followed by UFT/LV.

Abbreviations: AE, adverse event; NR, non-randomized; UFT/LV, uracil and tegafur plus leucovorin.

Supplementary Figure S1

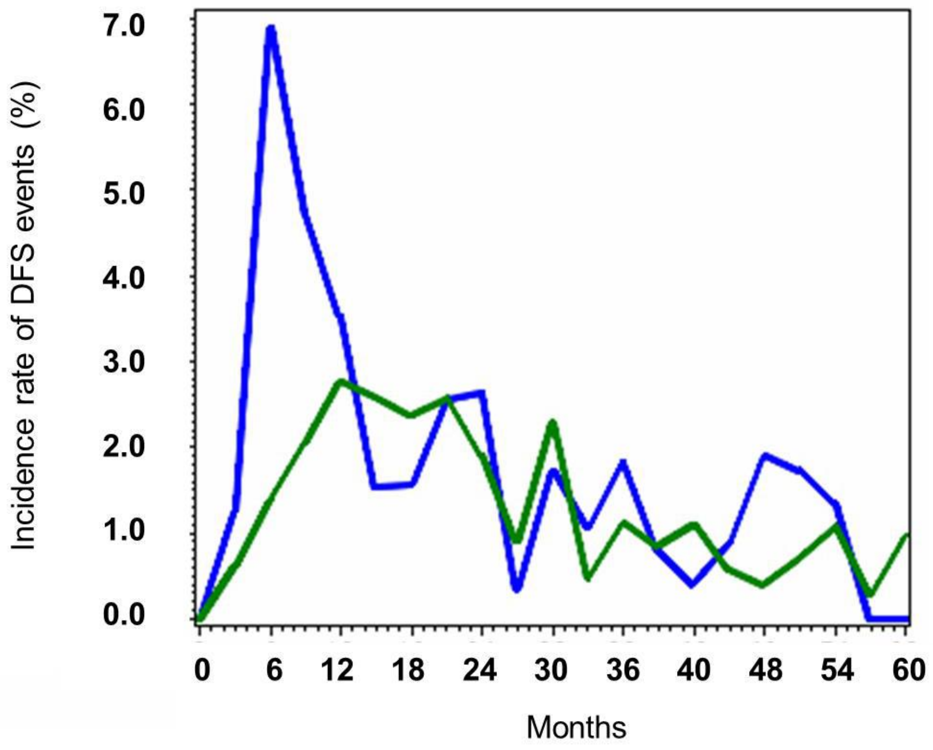

Incidence rate of disease-free survival events at 3-month intervals in propensity score-matched non-randomized patients with resected high-risk stage II colon cancer.

NR-Group S (blue): surgery alone; NR-Group U (green): surgery followed by UFT/LV treatment.

Abbreviations: DFS, disease-free survival; NR, non-randomized; UFT/LV, uracil and tegafur plus leucovorin.
